# A Bidirectional Permeability Assay for beyond Rule of 5 Compounds

**DOI:** 10.3390/pharmaceutics13081146

**Published:** 2021-07-27

**Authors:** Yunhai Cui, Cyril Desevaux, Ines Truebenbach, Peter Sieger, Klaus Klinder, Alan Long, Achim Sauer

**Affiliations:** 1 Department of Drug Discovery Sciences, Boehringer Ingelheim Pharma GmbH & Co. KG, 88397 Biberach, Germany; ines.truebenbach@boehringer-ingelheim.com (I.T.); peter.sieger@boehringer-ingelheim.com (P.S.); klaus.klinder@boehringer-ingelheim.com (K.K.); achim.sauer@boehringer-ingelheim.com (A.S.); 2 Exploratory Toxicology and Pharmacokinetics, Boehringer Ingelheim Animal Health, Athens, GA 30601, USA; cyril.desevaux@boehringer-ingelheim.com; 3 Chemistry and Lead Optimization, Boehringer Ingelheim Animal Health, Athens, GA 30601, USA; alan.long@boehringer-ingelheim.com

**Keywords:** permeability, physiological barrier, efflux, drug transporter, Lipinski’s Rule of 5, oral bioavailability, depsipeptides

## Abstract

Bidirectional permeability measurement with cellular models grown on Transwell inserts is widely used in pharmaceutical research since it not only provides information about the passive permeability of a drug, but also about transport proteins involved in the active transport of drug substances across physiological barriers. With the increasing number of investigative drugs coming from chemical space beyond Lipinski’s Rule of 5, it becomes more and more challenging to provide meaningful data with the standard permeability assay. This is exemplified here by the difficulties we encountered with the cyclic depsipeptides emodepside and its close analogs with molecular weight beyond 1000 daltons and cLogP beyond 5. The aim of this study is to identify potential reasons for these challenges and modify the permeability assays accordingly. With the modified assay, intrinsic permeability and in vitro efflux of depsipeptides could be measured reliably. The improved correlation to in vivo bioavailability and tissue distribution data indicated the usefulness of the modified permeability assay for the in vitro screening of compounds beyond the Rule of 5.

## 1. Introduction

**Figure 1:**
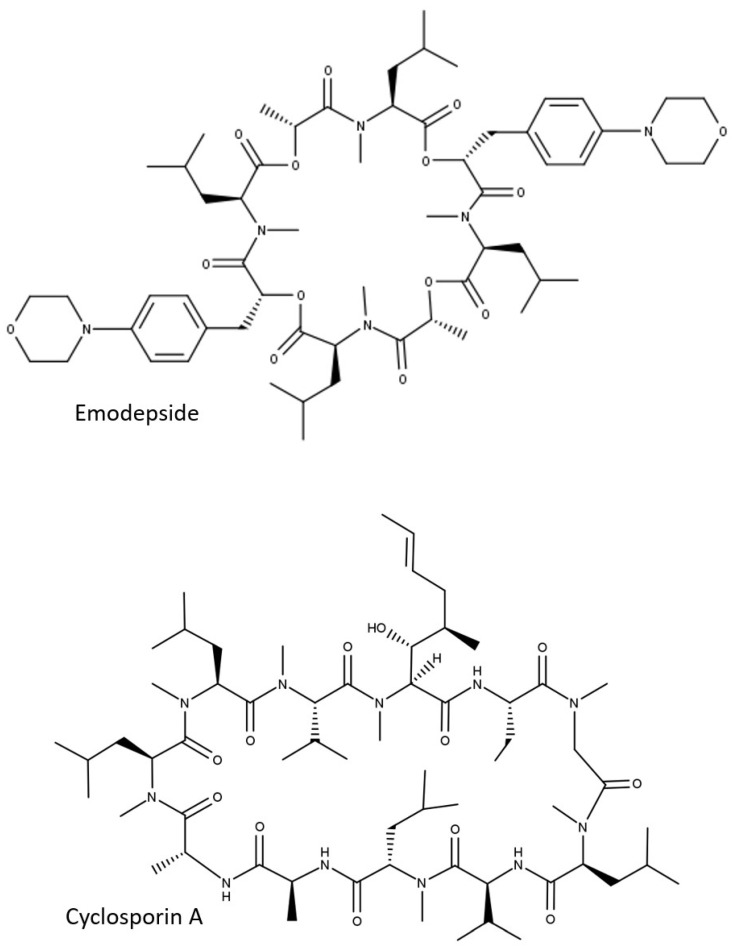
Two-dimensional structures of emodepside and cyclosporin A.

Permeability of drug substances across physiological barriers plays a major role in their absorption, distribution, and excretion. Bidirectional permeability measurement with cellular models grown on Transwell inserts is widely used in the pharmaceutical industry since it not only provides information about the passive permeability of a drug, but also about transport proteins involved in the active transport of drug substances across physiological barriers. The Caco-2 cell culture model, e.g., is considered the gold standard in vitro model for the study of drug absorption [1,2]. Lower expression levels or the absence of relevant transport proteins in Caco-2 cells accounts for the use of alternative models for barriers other than the gastrointestinal wall. Cell lines (e.g., Madin-Darby canine kidney cells, MDCK) with recombinant expression of human or animal MDR1 P-glycoprotein (P-gp, ABCB1) or breast cancer resistance protein (BCRP, ABCG2), e.g., are used to assess the ability of drug substances to cross the blood–brain barrier [3,4,5]. While these cellular models provided good in vitro–in vivo correlation with regard to intestinal absorption [6] or blood–brain barrier efflux [5], in the drug discovery programs of Boehringer Ingelheim Pharma (BI), we are encountering increasing numbers of compounds in drug discovery programs for which we are not able to provide meaningful permeability data using these models. One of the reasons for the difficulties in permeability measurement is the shift of the chemical space in research programs. While most of the marketed drugs follow the so-called Lipinski’s Rule of 5 (Ro5) [7], drug discovery programs deal more and more with difficult targets, such as protein–protein interactions. Large, flat binding sites can be better targeted with molecules beyond the Rule of 5 (bRo5), such as cyclic peptides [8]. Emodepside (Figure 1), e.g., a semisynthetic derivate of the metabolite of the fungus *Mycellia sterilia* PF1022A [9,10], is a cyclic octadepsipeptide [11,12] with molecular weight (MW) of 1119 daltons, originally developed as a topical and oral anthelmintic for veterinary use (Bayer AG, Leverkusen, Germany). Very recently, this drug was tested for the treatment of river blindness in humans and showed a dose-proportional increase in plasma exposure after oral administration [13], suggesting good permeability of emodepside to the gut wall. In addition, permeability of emodepside was also demonstrated by brain distribution data; though its brain levels in wildtype (WT) mice were not detectable, emodepside showed comparable exposure in brain and plasma in P-gp deficient mice, indicating its permeability to the blood–brain barrier and the protective role of P-gp [14]. Despite these in vivo observations, in vitro measurement of permeability with emodepside was not successful [15]. In one of our antiparasitic programs for animal health, we identified some close analogs of emodepside with excellent efficacy against heartworm in dog. These depsipeptides are also orally available and penetrant to the blood–brain barrier in P-gp deficient mice, as suggested by the strong differences in maximal tolerated doses in WT (>100 mg/kg) and P-gp deficient mice (ranging between 0.1 and 1 mg/kg). When tested in cellular permeability assays, these compounds were apparently poorly permeable. No meaningful P-gp efflux could be detected in the MDCK-MDR1 assay. The discrepancy between the in vivo observations and the in vitro results demonstrates the need for adaptation of the standard permeability assays currently used in the pharmaceutical industry for bRo5 compounds. The aim of this study is to identify the reason for the in vitro–in vivo disconnect regarding compound permeability and, if possible, modify the permeability assays accordingly.

## 2. Materials and Methods

### 2.1. Determination of LogP Values

The logP values (normal logarithm of partition coefficient between octanol and water) of the compounds were determined using an HPLC method described in the literature [16]:

A methanol–water gradient HPLC technique using a short octadecyl-polyvinyl alcohol (ODP) column (Shodex Asahipak ODP-50 4B, 5 µm material, 50 mm length, 4.6 mm inner diameter) was applied. Two internal standards (standard I: logP = 0.66; standard II: logP = 5.50) were included in each run. Since the correlation between logP and retention time is linear, the unknown logP can be determined using the logP and the retention time of the two standards and the retention time of the sample. As the compounds in this study were either neutral substances or weak bases, the logP determination was carried out at pH 11 to suppress ionization of the molecules.

### 2.2. In Vivo Pharmacokinetics and Tissue Distribution Studies in Mice

The in vivo experiments were conducted at contract research organizations in Europe (Nuvisan GmbH, Grafing, Germany; Evotec, Toulouse, France) and the United States (Avista Pharma, Durham, NC, USA) in compliance with the respective animal welfare regulations and approved by the responsible local authorities and committees. The animals were group housed under standard laboratory housing conditions with lights on in the morning. They had free access to water and standardized pelleted food.

Compounds were administered to adult male mice (strains: FVB/NCrl or CD-1) intravenously (2 mg/kg as a solution, maximal dose volume of 5 mL/kg) or orally (10 mg/kg as solution or 100 mg/kg as suspension in 0.5% Natrosol, dose volume of 10 mL/kg). Three animals were used per experiment. Serial plasma sampling and terminal tissue sampling were performed as previously described [5]. Drug concentrations in plasma and tissue homogenates were determined by HPLC-MS/MS (standard equipment: HPLC series 1000 or higher from Agilent, Santa Clara, CA, USA and mass spectrometers API 4000 or higher from AB Sciex, Toronto, ON, Canada). Measurement was conducted in multiple reaction monitoring (MRM) mode. Quantification was performed using external calibration. For the description of tissue distribution, tissue partition coefficients *K_p,brain_* (*K_p,br_*) and *K_p,brain/muscle_* (*K_p,br/mu_*) were calculated with the exposure in plasma (nM) and in brain or muscle (pmol/g tissue) as follows

(1) $$K_{p,br}=C_{brain}/C_{plasma}$$

(2) $$K_{p,br/mu}=C_{brain}/C_{muscle}$$

The partition coefficient *K_p,br/mu_* was used as a surrogate for the partition coefficient of unbound drug in brain *K_p,uu,brain_* [5].

### 2.3. Determination of Bidirectional Permeability in Caco-2 Cells and MDCK-MDR1 Cells

Caco-2 cells and MDCK-MDR1 cells below passage number 50 were used to produce large batches of assay-ready frozen cells (acCELLerate GmbH, Hamburg, Germany). Vials of assay-ready frozen cells were reconstituted in culture media and seeded directly onto Transwell inserts (#3379, Corning, Wiesbaden, Germany) without further expanding and passaging. Cells on Transwell inserts were cultured at 37 °C with 95% relative humidity and 5% CO_2_ for 14–21 days (Caco-2) or 9–10 days (MDCK-MDR1). Bidirectional permeability assays were performed as described previously [5,6]. Briefly, compounds were diluted in transport buffer (128.13 mM NaCl, 5.36 mM KCl, 1 mM MgSO_4_, 1.8 mM CaCl_2_, 4.17 mM NaHCO_3_, 1.19 mM Na_2_HPO_4_, 0.41 mM NaH_2_PO_4_, 15 mM 2-[4-(2-hydroxyethyl)piperazin-1-yl]ethanesulfonic acid (HEPES), 20 mM glucose, pH 7.4) containing 0.25% bovine serum albumin to a final concentration of 1 or 10 µM and added to the apical or basolateral (donor) compartment. Cells were incubated with the compounds for up to 2 h. Samples from the opposite (receiver) compartment were taken at different time points. Apparent permeability coefficients (*P_app,AB_*, *P_app,BA_*) were calculated as follows:

(3) $$P_{app,AB}=Q_{AB}/\left(C_{0}\times s\times t\right)$$

(4) $$P_{app,BA}=Q_{BA}/\left(C_{0}\times s\times t\right)$$

where *Q* is the amount of compound recovered in the receiver compartment after the incubation time *t*, *C*_0_ the initial compound concentration given to the donor compartment, and *s* the surface area of the Transwell inserts. Efflux ratio is calculated as the quotient of *P_app,BA_* (mean of duplicate) to *P_app,AB_* (mean of duplicate). Intrinsic apparent permeability coefficient (*P_app,Intrinsic_*) is calculated as mean of *P_app,AB_* and *P_app,BA_* [17]. In both Caco-2 and MDCK-MDR1 assays, the P-gp substrate apafant and one low permeable compound (BI internal reference, Papp ≈ 3 × 10^−7^ cm/s, no efflux) were included in every assay plate. In addition, Transepithelial electrical resistance (TEER) values were measured for each plate before the permeability assay. All three parameters (efflux of the reference substrates, *P_app_* values of the low permeable compound, and TEER values) were used to ensure the quality of the assays.

### 2.4. In Vitro Binding Assays

Binding of research compounds to plasma protein and tissue homogenates was determined using the equilibrium dialysis method as described previously [18]. Briefly, plasma, brain homogenate, and muscle homogenate were spiked with 1–10 µM of test compound and dialyzed in equilibrium dialysis cells (RED-device, Thermo Scientific/Pierce or Dianorm Equilibrium Device, Harvard Apparatus Holliston, MA, USA) against 100 mM potassium phosphate buffer pH 7.4 for 2–6 h at 37 °C. Fraction unbound in plasma (*f_u,plasma_*) was calculated as:

(5) $$f_{u,plasma}=C_{buffer}/C_{plasma}$$

where *C_plasma_* and *C_buffer_* are the plasma and buffer concentration, respectively. Unbound tissue concentration (*f_u,tissue_*) was calculated as:

(6) $$f_{u,tissue}=\left(1/D\right)/(1/f_{u,app}-1+1/D)$$

with *D* being the dilution factor and *f_u,app_* the observed fraction unbound in the homogenate incubations. All *f_u_* values were measured in triplicate.

Compound concentrations were measured using liquid chromatography-tandem mass spectrometry (HPLC-MS/MS) as previously described [18].

### 2.5. Dynamic Light Scattering Measurement

The aggregation behavior of the depsipeptides and cyclosporin A was determined by dynamic light scattering (DLS) using a NanoPartica SZ-100 (Horiba, Kyoto, Japan). All measurements were conducted at 25 °C with a detection angle of 173° and a measuring time of 120 s. Compound solutions were prepared in transport buffer (s. 2.3) at 0.1 µM, 1 µM, and 10 µM. For each sample, the measurement was recorded in triplicate. In DLS, the fluctuation of intensity in scattered light is correlated against short decay intervals (τ) and the intensity ACF (autocorrelation function) is obtained through the following mono-exponential equation:

G(2) (τ) = B + Bf exp (−2D_m_q^2^ τ) (7)

G(2)(τ): Measured amplitude autocorrelation function
B: So-called baseline
f: Instrument constant
D_m_: Particle diffusion coefficient
q: Scattering vector given by (4πn/λ)sin(θ/2)
τ: Delay time

A correlogram is generated where g2(τ) is plotted against the decay time [19].

### 2.6. Simulation of Unbound Intracellular Drug Concentrations in a Transwell Permeability Experiment

A simple three-compartment model (donor, cellular, and receiver compartments) was used for the simulation of the compound concentrations during incubation in a Transwell permeability experiment [17], with the modification that intracellular binding was also considered. The kinetic model was described by the following equations:

(8) dCu_donordt=2×Papp,intrinsic×S×(Cu_donor−Cu_cell)/Vdonor

(9) dCu_celldt=2×fu_cell×Papp,intrinsic×S×(Cu_donor−2×Cu_cell)/Vcell

(10) dCu_receiverdt=2×Papp,intrinsic×S×(Cu_cell−Cu_receiver)/Vreceiver

where *C_u_donor_*, *C_u_cell_*, and *C_u_receiver_* represent the unbound concentration of the test compound in the donor, cellular, and receiver compartment, *P_app,intrinsic_* the intrinsic apparent permeability coefficient across a cell monolayer, *S* the surface area of the apical and the basal plasma membrane, *V_donor_*, *V_cell_*, and *V_receiver_* the volume of the 3 compartments. Since the permeability assay is typically run under sink conditions—*C_u,receiver_* << *C_u,cell_*—Equation (9) can be further simplified as

(11) dCu_receiverdt=2×Papp,intrinsic×S×Cu_cell/Vreceiver

Parameters used for the simulation of a Transwell in 24-well format were: volume in donor and receiver compartment, 200 and 800 µL, respectively; surface area of cell monolayer facing the donor and receiver compartment, 0.33 cm^2^; volume of intracellular space, 0.33 µL (assuming a height of the monolayer of 10 µm); *P_app,Intrinsic_* of the compound, 1 × 10^−5^ cm/s. To simplify the model, no efflux transporters nor metabolic turnover were considered. A further assumption in this model is that the transition of the compound across the plasma membrane is much slower, compared with the binding kinetics in the cells, and thus represents the rate-limiting step of the transition process across a cellular monolayer. Simulation and visualization were conducted with Microsoft Excel.

## 3. Results

### 3.1. Depsipeptides Used in This Study

Physicochemical properties (MW, calculated and measured LogP) of depsipeptides and the reference cyclic peptide cyclosporin A are summarized in Table 1. With MW > 1000 and logP > 5, all of these peptides clearly belong to the bRo5 chemical space.

The oral bioavailabilities of the BI depsipeptides, determined by dosing 10 mg/kg of solution to CD-1 mice, are listed in Table 1. All BI depsipeptides are orally available, with BI-1 and -2 being slightly more available than BI-3 and 4. Oral bioavailability in mice was not measured for cyclosporin A nor emodepside in this study since the available data for human and rats [20,21] indicate a good bioavailability in these species.

Efflux at the blood–brain barrier in mice was evaluated by determining the tissue partition coefficient *K_p,br/mu_*, which was demonstrated to be a useful surrogate for *K_p,uu,brain_* for the evaluation of in vivo efflux at the blood–brain barrier [5]. Emodepside and its close analogs BI-1, BI-2, and BI-3 showed *K_p,br/mu_* < 0.1, indicating strong efflux at the blood–brain barrier (Table 1). For BI-4, only *K_p,br_* in WT mice was available. Due to the high similarity of the chemical structures of all depsipeptides in this study and due to the fact that for individual depsipeptides, *K_p,br_* and *K_p,br/mu_* are comparable, a similar *K_p,br_* and *K_p,br/mu_* was assumed for BI-4, suggesting high efflux of BI-4 at the blood–brain barrier.

### 3.2. In Vitro Permeability and Efflux Measured for Depsipeptides in Standard Assays

In contrast to its poor in vitro permeability as reported in [15], emodepside is highly permeable in standard cellular permeability assays (Table 2). Surprisingly, for all four BI depsipeptides derived from modifications of the side-chains of emodepside, no meaningful permeability data could be obtained in MDCK-MDR1 and Caco-2 cells. The data suggests very low permeability of these compounds. In most cases, compounds were below the detection limit in the receiver compartment. As the in vitro permeability data obviously contradicted the in vivo data obtained with BI depsipeptides, we suspected that the standard experimental conditions of the permeability assays were not compliant with the specific physicochemical properties of the depsipeptides. One possible explanation could be the compound loss due to nonspecific binding of the very lipophilic compounds to plastics, as hinted at by the low total recovery of BI-2 in the MDCK-MDR1 assay (Table 2). However, the addition of 2% BSA to the receiver compartment, which usually reduces nonspecific binding to plastic, did not improve the permeability of the compounds measured in these assays (data not shown).

### 3.3. Aggregation of Peptides in Aqueous Solutions

**Figure 2:**
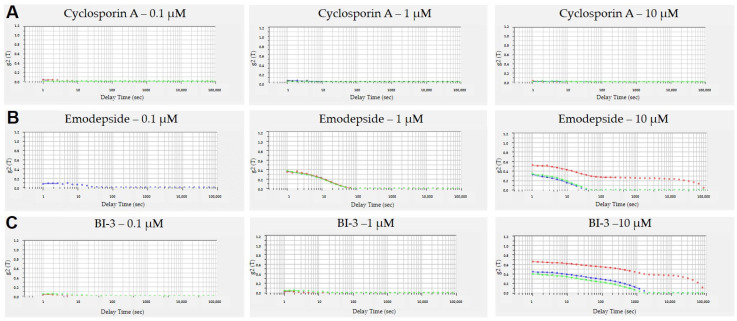
Time correlation functions of (**A**) cyclosporin A, (**B**) emodepside, (**C**) and BI-3 at 0.1 µM, 1 µM, and 10 µM. For each sample, the measurement was recorded in triplicate.

Another possible explanation for the apparent low in vitro permeability could be the aggregation of peptides in aqueous solutions resulting in low concentration of the monomers [23]. We thus measured their aggregation behavior with DLS. Aggregates create a colloid dispersion and scatter an incident laser; the intensity of the scattered light is detected by DLS. The aggregates are continuously mobile through Brownian diffusion and cause constructive and destructive interferences and hence, the intensity of scattered light fluctuates over time. The fluctuation of intensity in scattered light is correlated against short decay intervals (τ) and the intensity autocorrelation function (g2 (τ)) is obtained. The rate of decay of the autocorrelation function is used to extract aggregate size. Large aggregates diffuse slower than small particles do, and the correlation function decays at a slower rate. In the absence of aggregates, no autocorrelation signal can be observed [19]. Figure 2 depicts the time correlation functions of cyclosporin A, emodepside, and BI-3. Cyclosporin A (Figure 2A) does not aggregate at concentrations up to 10 µM, which is the standard concentration we used in our cellular permeability assays. Both depsipeptides emodepside and BI-3 showed concentration-dependent aggregation (Figure 2B,C), with emodepside showing a slightly higher tendency for aggregation. Encouraged by the lower aggregation at 0.1 and 1 µM, we repeated Caco-2 permeability measurement at 1 µM (0.1 µM was not possible due to the limit of quantification). As shown in Table 3, the intrinsic permeability (mean of *P_app,AB_* and *P_app,BA_*) of cyclosporin A and emodepside was comparable to that measured at 10 µM (Table 2), consistent with the concentration independence of permeability coefficients for passive diffusion. The higher efflux, measured at 1 µM for both compounds, on the other hand, was consistent with the nonlinear nature of active transport mediated by P-gp. Disappointedly, the apparent permeability of BI depsipeptides did not improve at all at the concentration of 1 µM.

### 3.4. Impact of Preincubation Time on Apparent In Vitro Permeability

**Figure 3:**
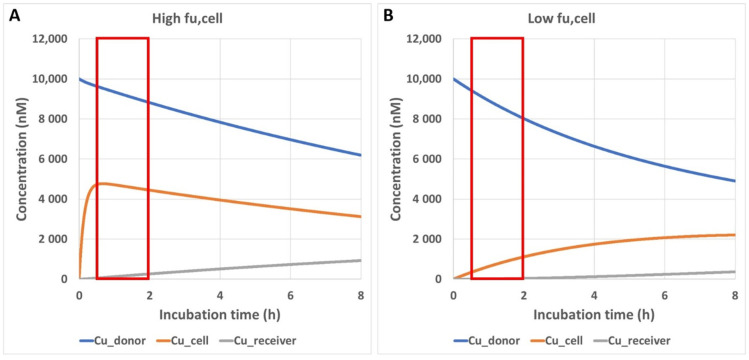
Simulated time course of the unbound compound concentrations in the donor compartment (*C_u_donor_*), in the cells (*C_u_cell_*), and in the receiver compartment (*C_u_receiver_*) in a permeability assay. Red boxes indicate the incubation period in the standard permeability assay which starts after 30 min of preincubation. Details of the model are described in Section 2.6. (**A**) *f_u,cell_* = 5%; (**B**) *f_u,cell_* = 0.1%.

Due to the high lipophilicity of the depsipeptides, incorporation into the membrane was hypothesized as a potential explanation for the apparent low permeability [15]. However, both cyclosporin A and emodepside are highly permeable in Caco-2 and MDCK-MDR1 cells, although they are only slightly less lipophilic compared with the other four depsipeptides (Table 1). Interestingly, we did find a remarkable difference between cyclosporin A and the depsipeptides: While free fraction (*f_u,plasma_*) in mouse and dog plasma was below 1% for all depsipeptides (the compounds were not detectable in dialysates), cyclosporin A had a rather high *f_u,plasma_* of 17% in human plasma [24]. In addition to the very tight plasma protein binding, the tissue binding of depsipeptides is similarly high, as suggested by the comparable *K_p,br_* and *K_p,br/mu_* (Table 2), e.g., *f_u,brain_* in rat brain homogenate was below 0.1% for emodepside. In contrast, a f_u,cell_ of 5.3% was reported for cyclosporin A in human blood cells [24]. The very low *f_u,tissue_* and *f_u,cell_* values for the cellular assays of the depsipeptides raises the question as to whether the standard incubation time of 1 to 2 h in most of the protocols of the cellular permeability assay could be too short. It is generally assumed that the unbound intracellular concentration of the test compound reaches a steady state within a much shorter time period compared with the total incubation time for most of the compounds [17]. Our simulations showed that a highly permeable compound with moderately high intracellular binding (*f_u,cell_* = 5%) needs about 30 min to reach steady state (Figure 3A). This is accounted for by a 30 min preincubation time in our standard assay protocol. Our simulations, however, also showed that for a highly permeable compound with very high intracellular binding (*f_u,cell_* = 0.1%), the time to steady state could be much longer than the standard preincubation time of 30 min (Figure 3B). Efflux transporters such as P-gp will further reduce the intracellular concentration of their substrates and prolong the time to steady state.

Based on these considerations, we made the following modifications to the assay conditions in the bidirectional permeability assays: compounds are diluted in culture media containing 5% fetal bovine serum (similar serum albumin concentration as in the transport buffer under standard conditions) to a final concentration of 1 µM and incubated with the cells grown on Transwell for 24 h. Subsequently, media were removed by aspiration and, after a wash with transport buffer without compounds, fresh transport buffer containing 1 µM compound was added. All subsequent steps were identical to the standard permeability assay. As shown in Table 4, the modified conditions for the Caco-2 assay led to an increase in P_app_ values and a slight reduction in efflux for emodepside (compare to Table 3). For BI depsipeptides, the modified assay not only led to measurable P_app_ values, but also allowed for differentiation between BI-1 and -2 vs. BI-3 and -4, with the latter two compounds showing lower oral availability in mice and lower permeability in Caco-2 cells. Similarly, the modified assay conditions also led to an improvement in data quality for the assay performed with MDCK-MDR1 cells. As shown in Table 5, in vitro efflux of emodepside fits very well to the in vivo efflux (efflux of 30.2 vs. 1/*K_p,br/mu_* of 29). The in vitro efflux of BI depsipeptides is comparable to that of emodepside and is consistent with the low *K_p,br_* or *K_p,br/mu_* in mouse (Table 1). In order to verify the role of P-gp in efflux measured in these cellular models, we performed the permeability measurement in the presence of 5 µM zosuquidar, a selective P-gp inhibitor. As shown in Table 4 and Table 5, efflux of all depsipeptides is completely inhibited in Caco-2 and MDCK-MDR1 cells by zosuquidar, indicating that all these depsipeptides are substrates of P-gp. Interestingly, Cyclosporin A, which has a much higher *f_u,cell_* than depsipeptides, showed rather comparable permeability with the standard and the modified assay conditions, suggesting the need for a prolonged preincubation time only for compounds with very low *f_u,cell_*.

Since the examples showed above are all bRo5 compounds, we asked ourselves whether the prolonged preincubation time would affect the permeability data for Ro5 compounds in general. Apafant e.g., a P-g substrate which is used for quality control in every assay run, has no violation of Ro5 (MW 456, cLogP 1.1, H-donor 0, H-acceptor 5). The permeability of apafant in Caco-2 assay with standard and modified conditions are very similar (*P_app,AB_* 2.3 × 10^−6^ vs. 2.6 × 10^−6^ cm/s, efflux 15.9 vs. 13.8). In the modified Caco-2 assay, the addition of zosuquidar inhibited completely P-gp efflux and resulted in a *P_app,AB_* of 13 × 10^−6^ cm/s, which is highly comparable to the intrinsic permeability of apafant in the standard assay (14 × 10^−6^ cm/s). Importantly, the low permeable compound (MW 376.5, cLogP 0.6, H-donor 1, H-acceptor 3), which is used as quality control in all our permeability assays, demonstrated similarly low permeability under the standard and modified conditions (*P_app,intrinsic_* ~ 0.3 × 10^−6^ cm/s), indicating an intact monolayer under the conditions with prolonged preincubation time.

**Figure 4:**
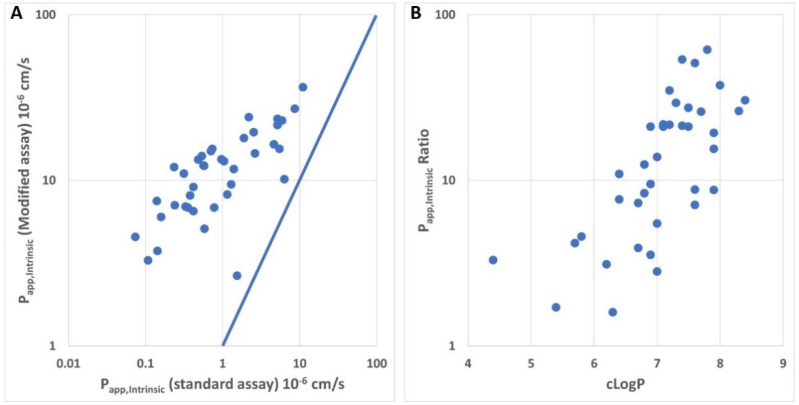
Comparison between the apparent permeability measured with the standard and the modified MDCK-MDR1 assay for a chemical series (N = 39). The standard permeability assay was performed as described in Section 2.3. The modified permeability assay with prolonged preincubation time is described above. All *P_app_* values represent mean values of duplicates. *P_app,Intrinsic_* ratio is calculated by dividing *P_app,Intrinsic_* from the modified assay by *P_app,Intrinsic_* from the standard assay. (**A**) *P_app,Intrinsic_* values from the modified assay were plotted against *P_app,Intrinsic_* values from the standard assay. Solid line shows 1:1 correlation. (**B**) Ratios between *P_app,Intrinsic_* values from standard and modified assays were plotted against cLogP.

We next evaluated whether the size of the molecules (MW) or the lipophilicity is the major driver for the poor apparent permeability in the standard assay. Figure 4 presents a chemical series from one of our drug discovery projects. All compounds (N = 39) are in a narrow range of MW between 460 and 550 (Table S1). Despite the rather small size, the compounds are highly lipophilic with cLogP between 4.4 and 8.4 (MoKa 2.6.4). Interestingly, there is a clear shift towards higher apparent permeability measured with the modified assay compared with the values measured with the standard assay (Figure 4A). It was even more interesting to note that there is a striking correlation between the shift and the lipophilicity (Figure 4B).

## 4. Discussion

With chemistry expanding into the bRo5 space in pharmaceutical research, standard in vitro ADME (absorption, distribution, metabolism, and excretion) assays often encounter difficulties in providing meaningful data to guide compound optimization, as exemplified here by our difficulties in measuring the permeability of cyclic depsipeptides. Different explanations have been proposed in the literature, such as incorporation into lipid bilayer [15], aggregation in aqueous solution [23], and compound loss due to nonspecific binding to the plasticware [25]. Our results here, however, did not agree with these explanations. The strong discrepancy between the permeability of emodepside, measured with the parallel artificial membrane permeability assay (PAMPA), and with Caco-2 or MDCK-MDR1 cells suggests that incorporation into lipid bilayer should not be a major hurdle for permeability across plasma membranes; in cellular permeability models, emodepside has to cross the plasma membrane twice. For highly lipophilic compounds, PAMPA does not seem to be a suitable alternative to the cellular permeability models. Although we did observe concentration-dependent aggregation of emodepside and BI-3 in DLS measurement, reducing the concentration of the incubation buffer to 1 µM did not improve the permeability measurement, suggesting low impact of aggregation on the apparent permeability. As the addition of 2% BSA to the receiver compartment did not improve the permeability either, we think that compound loss due to nonspecific binding to plasticware is most likely not the major reason for the very poor apparent permeability of depsipeptides. Interestingly, Huth et al. did show improvement in apparent permeability by adding 2% BSA to the receiver compartment [25]. In addition to the reduction in compound loss due to binding to plasticware, the authors argued that highly lipophilic compounds tend to have extensive partition into cells and thus need more time to achieve intracellular steady-state concentrations. BSA in the receiver compartment would reduce the cellular partition and accelerate the exit of the compounds. Though we agree with the extensive partition of highly lipophilic compounds into cells, we think that the addition of BSA would only improve the apparent permeability if the concentration of the compounds in the receiver compartment increases too fast to maintain sink condition, i.e., negligible concentration in the receiver compartment, and thus negligible back diffusion of the compounds into cells, during the incubation period of the measurement. For most of the compounds tested here, however, poor apparent permeability means very low compound concentration in the receiver compartment. Sink condition thus cannot be further improved by the addition of BSA into the receiver compartment.

As discussed by Huth et al., we do think that the time taken to reach steady-state concentration in the cells has an important impact on the apparent permeability measured under the standard conditions. Our simulation shows that a highly permeable compound with a *f_u,cell_* of 5% needs about 30 min to achieve steady state in the cells (Figure 3A); one example of such a compound is cyclosporin A. Despite the high lipophilicity (LogP 5.5), cyclosporin A has a rather moderate cellular partition [24]. This explains why the compound behaves rather normally in the permeability assays with a standard preincubation time of 30 min. Depsipeptides with *C_u,cell_* < 0.1% would need much longer to achieve steady state in the cells according to our simulation (Figure 3B). The improvement in the apparent permeability of these compounds with prolonged preincubation time is consistent with this hypothesis. Interestingly, emodepside, in contrast to the other depsipeptides, also showed good permeability in the standard assays. This could be explained by the lower lipophilicity of emodepside (0.5–0.8 log unit lower, Table 1). As shown in Figure 4B, there is a striking correlation between the increase in apparent permeability with longer preincubation time and cLogP. Even though we were not able to measure *f_u,cell_* for all depsipeptides tested in this study, emodepside, with the lowest logP, might have a higher *f_u,cell_* compared with the other depsipeptides, which allowed for a measurable permeability in the standard assays. Nevertheless, prolonged preincubation also led to higher apparent permeability for emodepside, suggesting that the standard preincubation time of 30 min is not sufficient for emodepside. The simulation shown in Figure 3B also raises the question as to what the optimal preincubation time for bRo5 compounds would be. We chose the preincubation time of 24 h for two reasons: (1) We are aware that compounds with different *f_u,cell_* would need different preincubation times. However, in a screening setting, it is not feasible to adapt the preincubation time for different compounds. (2) Consequently, the preincubation time should be long enough to provide meaningful permeability data for the majority of our research compounds, but short enough to avoid any possible artefacts associated with long preincubation times. Our first evaluation showed that 4–6 h would not be sufficient for depsipeptides (not shown). The preincubation time of 24 h that we are currently using seems to provide reasonable in vitro–in vivo correlation regarding blood–brain barrier efflux and oral availability for tested compounds. On the other hand, for the Ro5 compounds tested so far, similar data were obtained to when the standard assay conditions were used. In addition, efflux measured in the modified assay with prolonged preincubation was completely inhibited by the P-gp inhibitor zosuquidar. We are thus confident that the modified assay indeed provides improved data quality, especially for bRo5 compounds. It is important to note that the nature of the cellular distribution of the depsipeptides, i.e., binding to lipids or to proteins, is not relevant for the permeability measurement as long as the transition across the plasma membrane is the rate-limiting step during the transcellular flow of the compounds, which was the basic assumption for the simulation shown in Figure 3. Fast equilibrium between aqueous phase and partition into lipid bilayers was also used by more comprehensive models for the kinetic description of transport across confluent monolayer [26], because the binding kinetics of amphiphilic compounds to proteins and lipid bilayers seems to be very fast [27]. Though we do not have any proof as to whether this also holds true for bRo5 compounds, restrictive binding to the cellular components, i.e., the *k_off_* being the rate-limiting step, would not agree with an increase in the apparent permeability by an increase in the preincubation time.

A question associated with the long preincubation time is, however, the physiological relevance of the long preincubation time compared with the much shorter time frame for intestinal absorption. This could potentially be explained by the different ratios between surface area and cellular volume for in vitro models and for intestinal mucosa. Caco-2 cells originated from human colon, which is known to have less surface area enlargement due to less microvilli structures compared with small intestines [28]. The larger absorptive surface area in small intestines would shorten the time to steady state in the mucosa epithelial cells and accelerate the absorption in vivo. In addition to the intestinal absorption, time to intracellular steady state could also have an impact on the distribution of bRo5 compounds into target cells, especially on the time for the compounds to achieve effective unbound concentration in the cells. Interestingly, for a few compounds with strong improvement of apparent permeability in the modified assay, we also observed increasing potency against an intracellular target with increasing incubation time in pharmacological assays using human and mouse whole blood (data now shown). This observation suggests that the time to steady state of intracellular concentration for such compounds could be much longer than for Ro5 compounds and underlines the relevance of the long incubation time.

One possible issue associated with the long preincubation time could be cytotoxicity of the test compounds. Cytotoxicity could lead either to leaky monolayer of the cells on Transwell inserts, resulting in artificially high permeability, or to reduction in transporter activities, resulting in underestimation of efflux. Though neither was observed for compounds tested up to now, cytotoxicity could not be excluded for future compounds. Because cytotoxicity is usually closely monitored in pharmacological screening assays which occur before the permeability assays, the risk of cytotoxicity in permeability assays is relatively low.

In summary, we described here a modified permeability assay with prolonged preincubation time. The modified assay conditions resulted in improved data quality for bRo5 compounds regarding cellular permeability and involvement of efflux transporters. This assay can be easily integrated into our standard permeability assay platform and provides sufficient throughput to support our drug discovery projects working in the chemical space beyond the Rule of 5.

## Figures and Tables

**Table 1:** Cyclopeptides used in this study.

Compound	MW	cLogP ^1^	LogP ^2^	Oral Availability in Mice (%) ^3^	*K_p,br_* in WT Mice	*K_p,br/mu_* in WT Mice
Cyclosporin A	1202.6	6.6	5.5	n.d.	n.d.	~0.1 ^4^
Emodepside	1119.4	4.3	5.6	n.d.	0.05 ^5^	0.034 ^5^
BI-1	1181.5	6.5	6.4	33	0.020 ^5^	0.038 ^5^
BI-2	1177.4	5.7	6.1	28	0.13 ^5^	0.082 ^5^
BI-3	1121.2	6.4	6.1	14	0.011 ^5^	0.025 ^5^
BI-4	1153.2	7.0	6.3	10	0.023 ^5^	n.d.

n.d.: Not determined. ^1^ cLogP is calculated using the software MoKa version 2.6.4 (Molecular Discovery, Borehamwood, Hertfordshire, United Kingdom). ^2^ LogP is determined as described in Section 2.1. ^3^ Oral availability determined with an intravenous bolus dose of 2 mg/kg and an oral dose of 10 mg/kg. ^4^ Estimated with data from [22]. ^5^ *K_p,br_* and *K_p,br/mu_* determined with an oral dose of 10 or 100 mg/kg, as described in Section 2.2.

**Table 2:** Bidirectional permeability measured in MDCK-MDR1 cells and Caco-2 cells. Data are mean values of duplicates from standard permeability assays (see Section 2.3). Final concentration of compounds in donor compartment was 10 µM. Highly permeable compounds metoprolol and propranolol are shown as reference.

Compound	MDCK-MDR1			Caco-2		
	*P_app,AB_* (10^−6^ cm/s)	*P_appBA_* (10^−6^ cm/s)	Efflux	*P_app,AB_* (10^−6^ cm/s)	*P_appBA_* (10^−6^ cm/s)	Efflux
Metoprolol	67.7	68.5	1.0	54.3	41.0	0.8
Propranolol	38.4	32.6	0.8	79.0	69.5	1.1
Cyclosporin	7.6	68.2	10.8	22.2	17.9	0.8
Emodepside	3.6	34.1	9.5	27.4	16.4	0.8
BI-1	<0.3	1.2	n.c.	<0.12	0.12	n.c.
BI-2	Low recovery		n.c.	<0.47	1.73	n.c.
BI-3	<0.02	0.06	n.c.	<0.14	0.05	n.c.
BI-4	<0.1	<0.06	n.c.	<0.06	0.02	n.c.

n.c.: Not calculated.

**Table 3:** Bidirectional permeability measured in Caco-2 cells. Data are mean values of duplicates from standard permeability assays. Final concentration of compounds in donor compartment was 1 µM.

Compound	Caco-2		
	*P_app,AB_* (10^−6^ cm/s)	*P_appBA_* (10^−6^ cm/s)	Efflux
Cyclosporin	7.2	26.4	3.7
Emodepside	8.8	36	4.1
BI-1	<0.53	<0.23	n.c.
BI-2	BLQ	BLQ	n.c.
BI-3	<1.2	0.65	n.c.
BI-4	<0.036	<0.014	n.c.

n.c.: Not calculated.

**Table 4:** Modified bidirectional permeability assay with Caco-2 cells. Data are mean values of duplicates. Final concentration of compounds in donor compartment was 1 µM.

Compound	Caco-2			Caco-2 in the Presence of Zosuquidar		
	*P_app,AB_* (10^−6^ cm/s)	*P_app,BA_* (10^−6^ cm/s)	Efflux	*P_app,AB_* (10^−6^ cm/s)	*P_app,BA_* (10^−6^ cm/s)	Efflux
Cyclosporin	10	44	4.4			
Emodepside	48	110	2.3	60	35	0.7
BI-1	5	29	5.8	4.8	5.0	1.0
BI-2	13	43	3.3	26	23	0.9
BI-3	0.9	8.2	9.3	2.0	2.4	1.2
BI-4	0.2	4.2	23.3	1.8	1.3	0.7

**Table 5:** Modified bidirectional permeability assay with MDCK-MDR1 cells. Data are mean values of duplicates. Final concentration of compounds in donor compartment was 1 µM.

Compound	MDCK-MDR1			MDCK-MDR1 in the Presence of Zosuquidar		
	*P_app,AB_* (10^−6^ cm/s)	*P_app,BA_* (10^−6^ cm/s)	Efflux	*P_app,AB_* (10^−6^ cm/s)	*P_app,BA_* (10^−6^ cm/s)	Efflux
Cyclosporin	2.1	62	29.5	28	13	0.5
Emodepside	6.3	190	30.2	56	39	0.7
BI-1	0.7	30	45.5	4.4	3.7	0.8
BI-2	2.4	47	19.6	9.4	7.3	0.8
BI-3	<0.037	3.1	n.c.	1.8	1.6	0.9
BI-4	0.3	12.4	47.5	1.6	1.4	0.9

n.c.: Not calculated.

## Data Availability

The data presented in this study are available in Supplementary Materials.

## Supplementary Materials

The following are available online at https://www.mdpi.com/article/10.3390/pharmaceutics13081146/s1, Table S1: Compounds used for the analyses shown in Figure 4.
