# Supplementary material for: A Bidirectional Permeability Assay for beyond Rule of 5 Compounds
Source: Pharmaceutics. 2021 Jul 27;13(8):1146. doi: 10.3390/pharmaceutics13081146 (PMC8400635; doi:10.3390/pharmaceutics13081146)
Supplement: Supplementary file 1 [file pharmaceutics-13-01146-s001.zip › pharmaceutics-1297219-supplementary.pdf]

pharmaceutics

Supplementary Materials: A Bidirectional Permeability Assay for Beyond Rule of 5 Compounds

Yunhai Cui, Cyril Desevaux, Ines Truebenbach, Peter Sieger, Klaus Klinder, Alan Long and Achim Sauer

Table S1. Compounds used for the analyses shown in Figure 4.

Compound	cLogP	MW	$P_{app,Intrinsic}$ (10^{-6} cm/s) Standard assay	$P_{app,Intrinsic}$ (10^{-6} cm/s) Modified assay	$P_{app,Intrinsic}$ (modified assay)/ $P_{app,Intrinsic}$ (standard assay)
BI_5	4.4	523.5	11.1	36.5	3.3
BI_6	5.4	538.4	1.6	2.7	1.7
BI_7	5.7	500.9	5.2	21.5	4.2
BI_8	5.8	496.4	5.2	23.5	4.6
BI_9	6.2	494.5	8.7	27.0	3.1
BI_10	6.3	550.4	6.4	10.2	1.6
BI_11	6.4	462.5	2.2	24.0	10.9
BI_12	6.4	514.9	2.6	19.5	7.6
BI_13	6.7	511.4	5.9	23.0	3.9
BI_14	6.7	515.8	1.3	9.5	7.3
BI_15	6.8	464.5	1.0	13.0	12.4
BI_16	6.8	531.4	1.4	11.7	8.4
BI_17	6.9	482.5	0.6	12.2	21.0
BI_18	6.9	493.5	1.9	18.0	9.5
BI_19	6.9	495.4	4.7	16.5	3.5
BI_20	7	482.5	5.5	15.5	2.8
BI_21	7	492.4	2.7	14.5	5.5
BI_22	7	500.5	1.0	13.4	13.8
BI_23	7.1	500.5	0.6	12.3	21.8
BI_24	7.1	527.4	0.7	15.5	21.1
BI_25	7.1	513.4	0.7	15.0	21.1
BI_26	7.2	550.3	0.3	11.0	34.8
BI_27	7.2	509.4	0.4	9.1	21.7
BI_28	7.3	529.9	0.2	7.1	29.4
BI_29	7.4	499.0	0.4	8.1	21.3
BI_30	7.4	527.4	0.1	7.5	53.6
BI_31	7.5	494.4	0.3	7.0	21.1
BI_32	7.5	495.4	0.5	13.3	27.4
BI_33	7.6	517.0	1.2	8.2	7.1
BI_34	7.6	513.8	0.8	6.9	8.8
BI_35	7.6	528.9	0.2	12.0	51.1
BI_36	7.7	549.3	0.5	14.0	25.9
BI_37	7.8	508.4	0.1	4.6	61.5
BI_38	7.9	497.4	0.4	6.5	15.5
BI_39	7.9	533.4	0.6	5.1	8.7
BI_40	7.9	533.4	0.4	6.9	19.3
BI_41	8	533.4	0.2	6.0	37.5
BI_42	8.3	542.9	0.1	3.8	26.2
BI_43	8.4	549.9	0.1	3.3	30.4
